# Supplementary figures and images for: tRF-1:30-Gly-CCC-3 inhibits thyroid cancer via binding to PC and modulating metabolic reprogramming
Source: Life Sci Alliance. 2023 Dec 11;7(3):e202302285. doi: 10.26508/lsa.202302285 (PMC10713435; doi:10.26508/lsa.202302285)

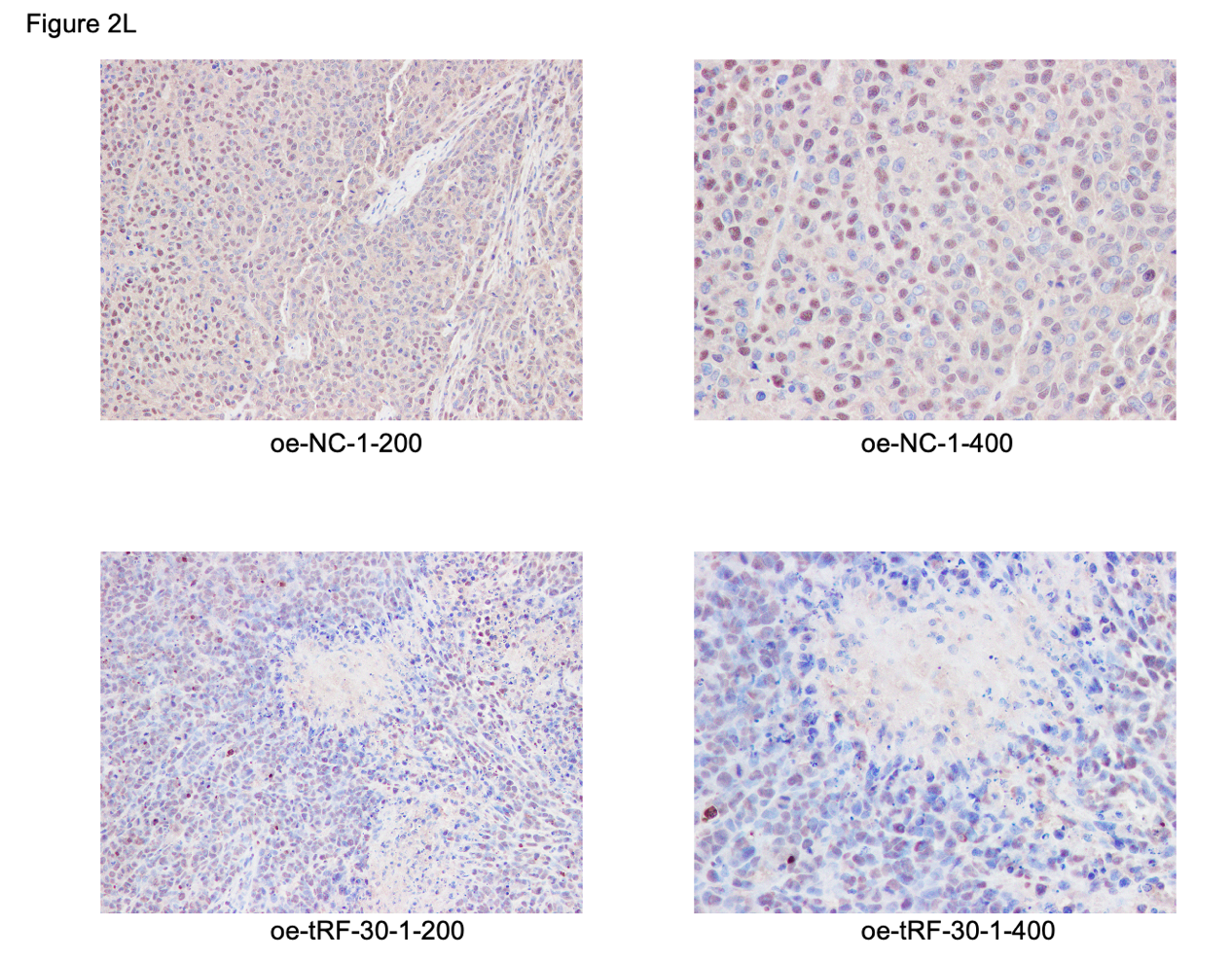

Supplement: Supplementary file 3 [file LSA-2023-02285_SdataF2.docx]

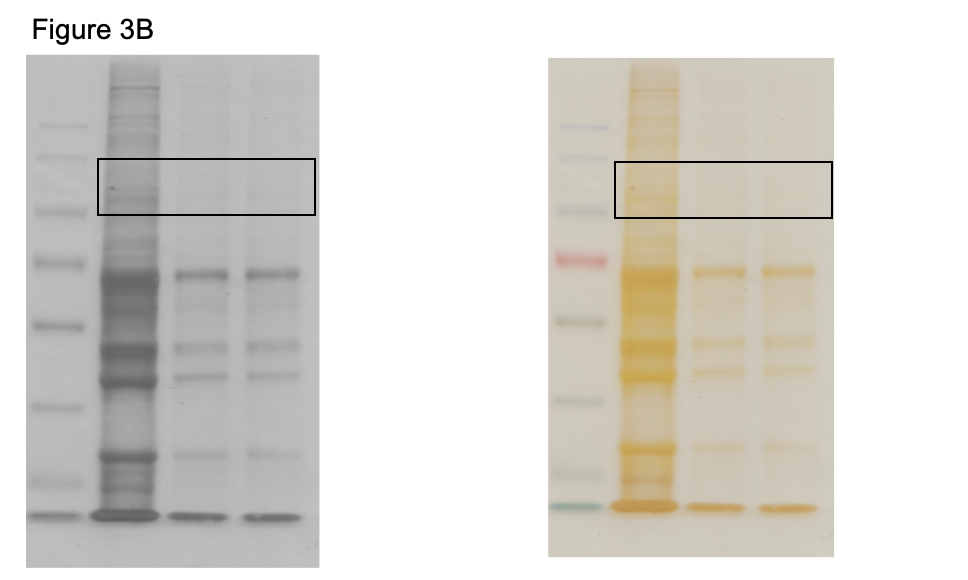


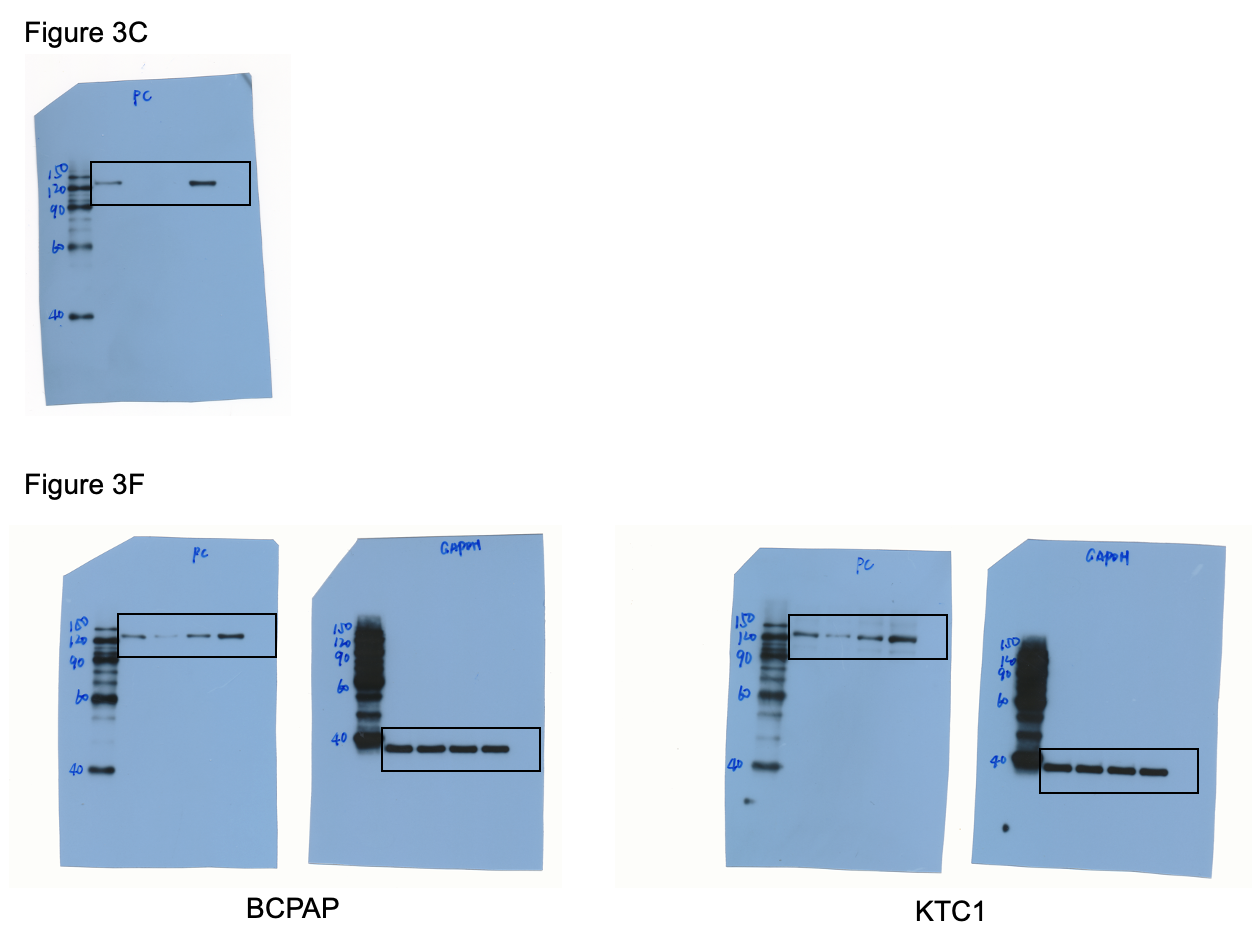


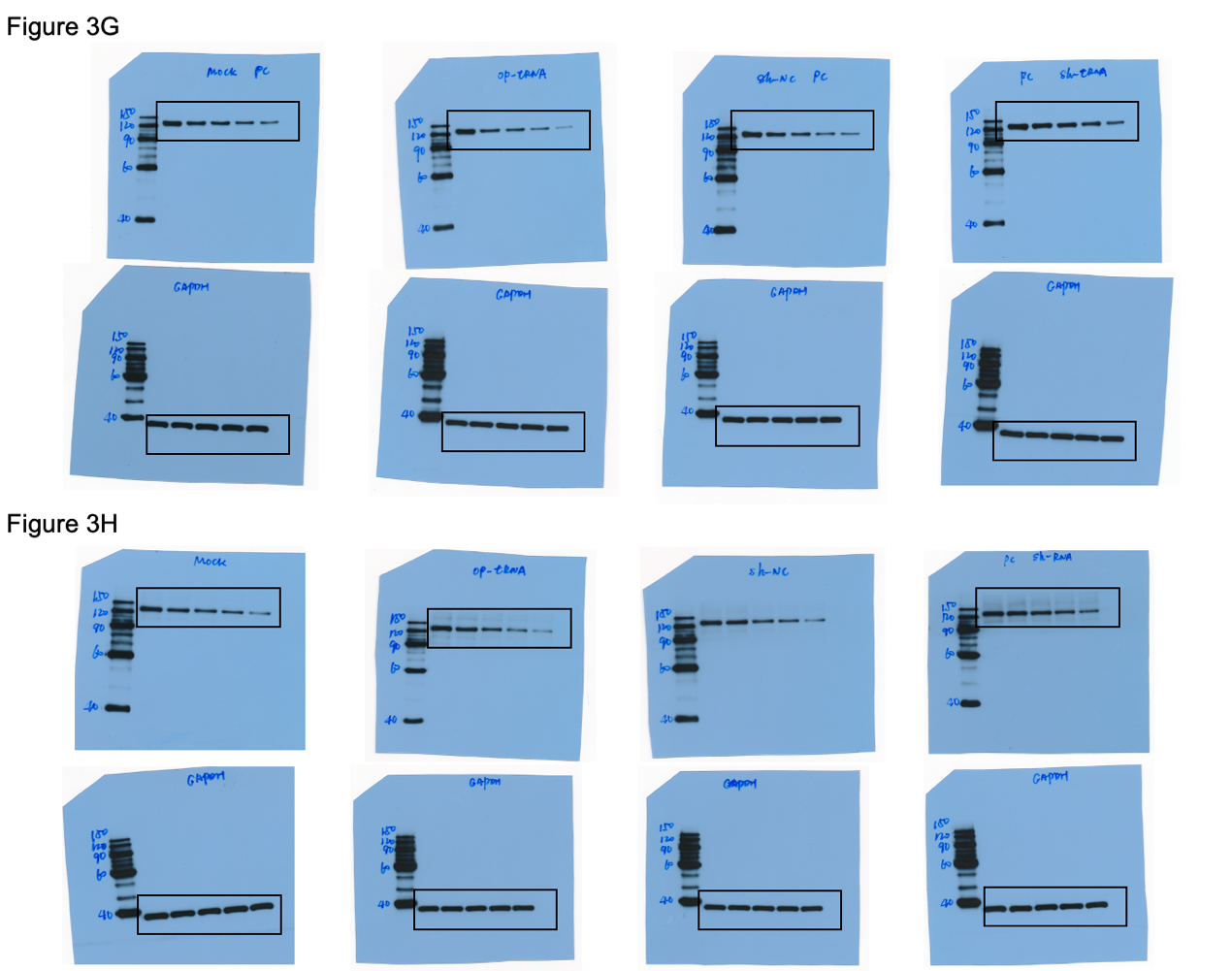

Supplement: Supplementary file 4 [file LSA-2023-02285_SdataF3.docx]

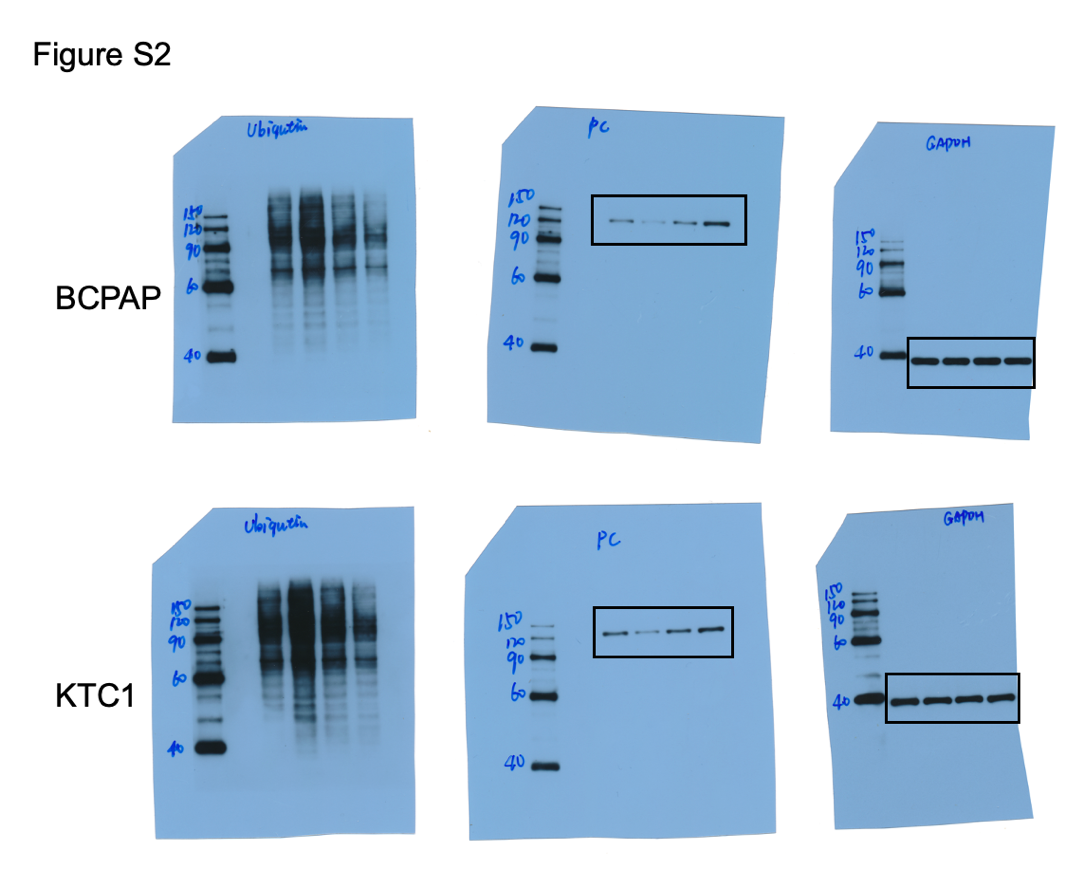

Supplement: Supplementary file 6 [file LSA-2023-02285_SdataFS2.docx]

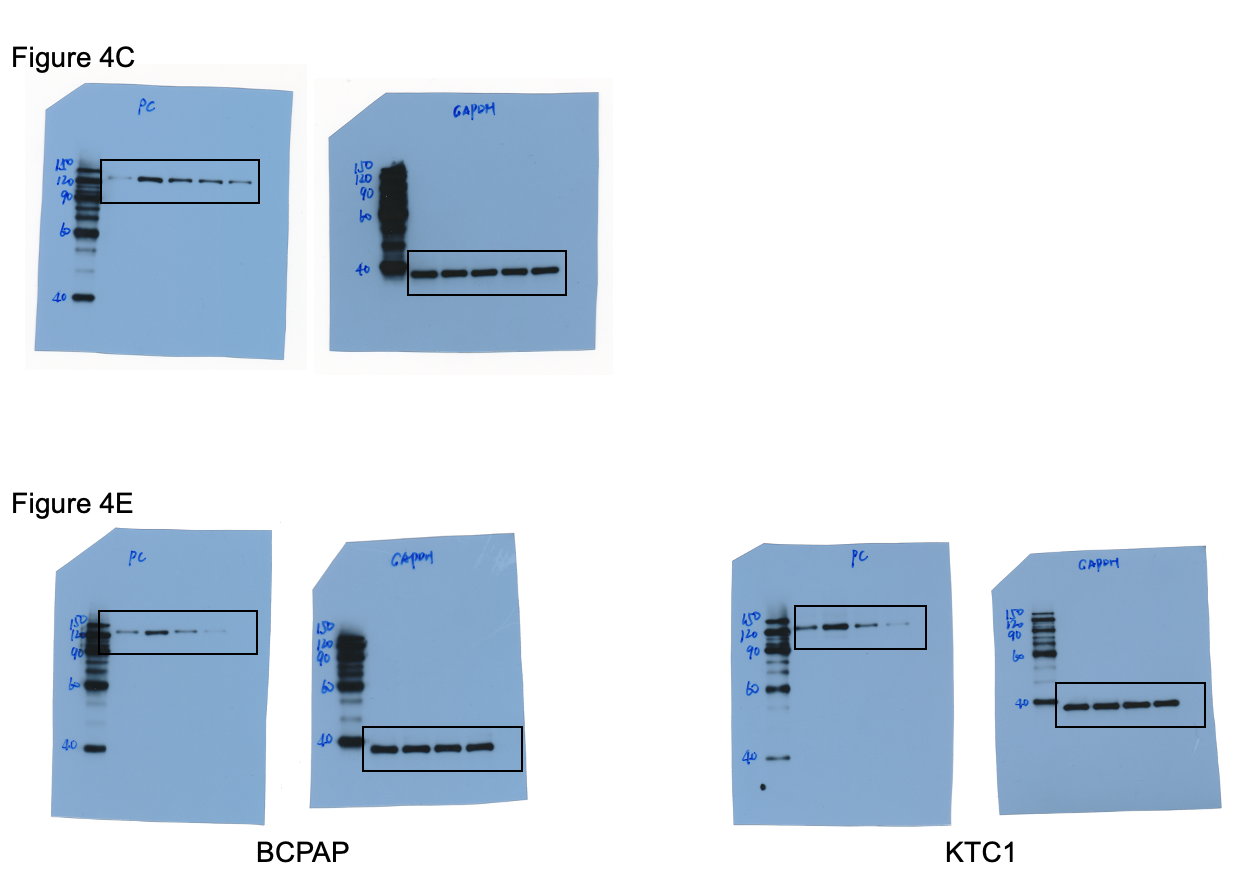

Supplement: Supplementary file 7 [file LSA-2023-02285_SdataF4.docx]

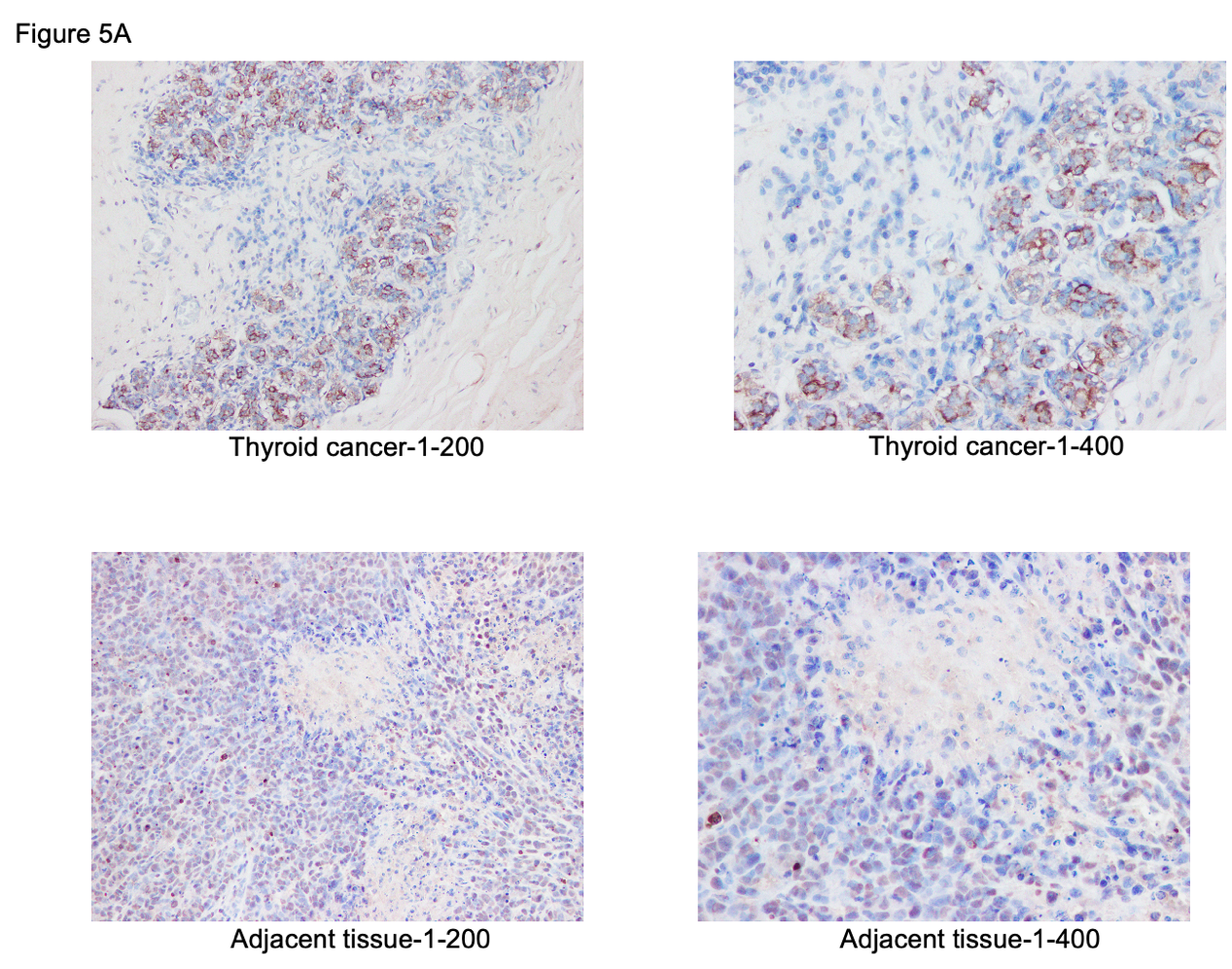


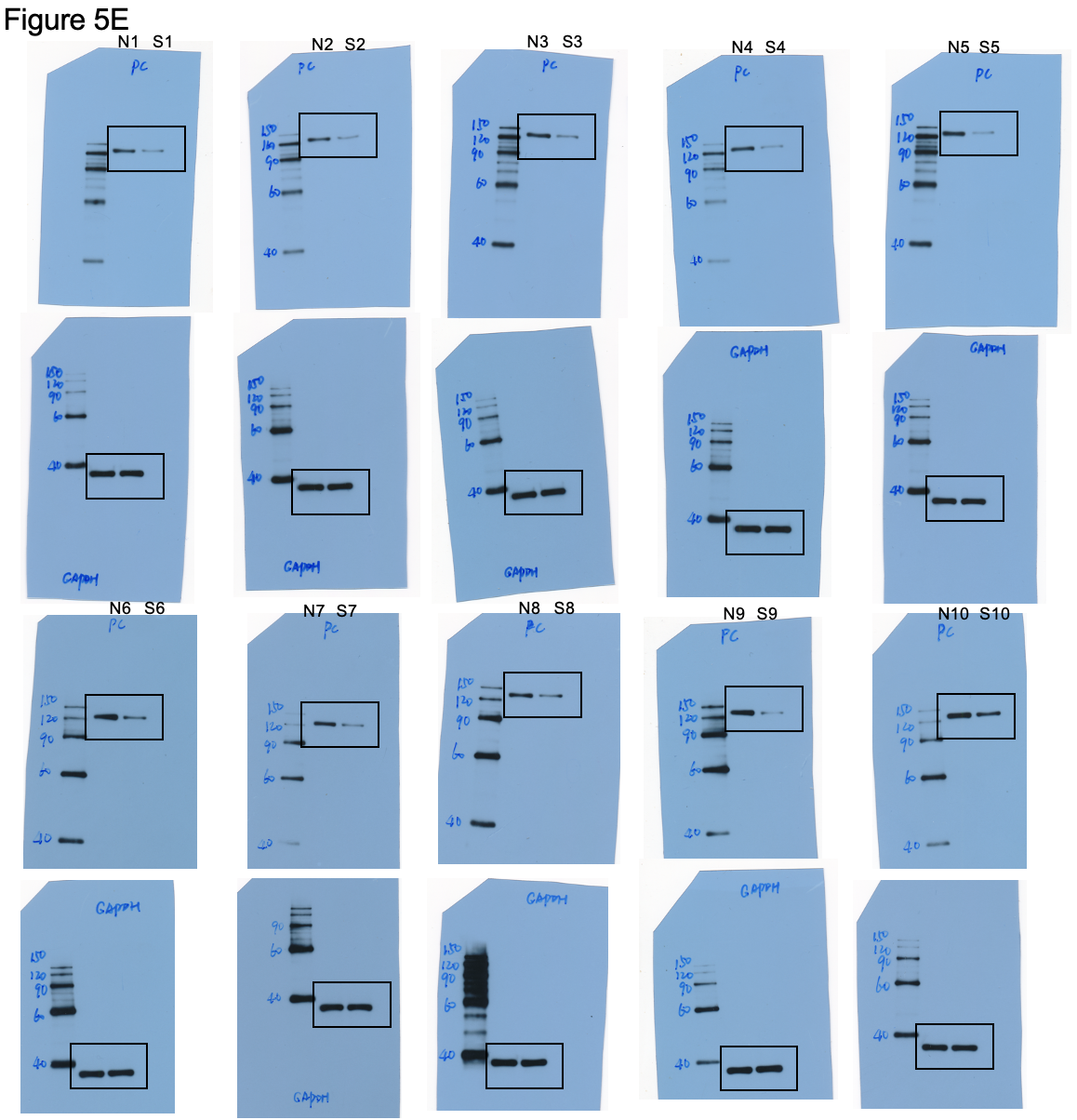

Supplement: Supplementary file 8 [file LSA-2023-02285_SdataF5.docx]
