# Supplementary material for: tRF-1:30-Gly-CCC-3 inhibits thyroid cancer via binding to PC and modulating metabolic reprogramming
Source: Life Sci Alliance. 2023 Dec 11;7(3):e202302285. doi: 10.26508/lsa.202302285 (PMC10713435; doi:10.26508/lsa.202302285)
Supplement: Supplementary file 10 [file LSA-2023-02285_TableS3.docx]

Table S3 Real-time PCR primers used in the study.

| Gene | Primer sequences (5′ to 3′) |
| --- | --- |
| *actin* | F: 5′-GTCCACCGCAAATGCTTCTA-3′ |
|  | R: 5′-TGCTGTCACCTTCACCGTTC-3′ |
| *U6* | F: 5′-GCTTCGGCAGCACATATACTAAAAT-3′ |
|  | R: 5′-CGCTTCACGAATTTGCGTGTCAT-3′ |
| *Pc* | F: 5′-GGACCAGTATGGGAACATCCT-3′ |
|  | R: 5′-GTTGACCTCGATGAAGTAGTGC-3′ |
| *tRF-30* | F: 5′-ATCGCATTGGTGGTTCAATGG-3′ |
|  | R: 5′-TGTGCTCTTCCGATCTGCGA-3′ |
