# Supplementary material for: tRF-1:30-Gly-CCC-3 inhibits thyroid cancer via binding to PC and modulating metabolic reprogramming
Source: Life Sci Alliance. 2023 Dec 11;7(3):e202302285. doi: 10.26508/lsa.202302285 (PMC10713435; doi:10.26508/lsa.202302285)
Supplement: Supplementary file 11 [file LSA-2023-02285_TableS4.docx]

Table S4 Oligonucleotide sequences used in the cell transfection.

| **GROUP** | **OLIGONUCLEOTIDE SEQUENCES (5′ to 3′)** |
| --- | --- |
| oe-tRF-30  oe-NC | GCAUUGGUGGUUCAAUGGUAGAAUUCUCGC  UUGCAACUUGGAGGGGUGUAACUCUUCUGA |
| sh-PC  sh-NC | CUACAAAAUUGGUGGUAUUTT  GTGTCGAGCAAGCTTGGTAGGTCAT |
| sh-tRF-30 | GCAUUGGUGGUUCAAUGGUAGAAUU |
| sh-NC | AGUGGAUAGUUUAUUCGCGGAAUGU |
| oe-PC | Purchased from Qiagen containing the coding sequence (NM_000920) |
